# A congruent topology for deep gastropod relationships

**DOI:** 10.1098/rspb.2018.2776

**Published:** 2019-03-13

**Authors:** Tauana Junqueira Cunha, Gonzalo Giribet

**Affiliations:** Museum of Comparative Zoology, Department of Organismic and Evolutionary Biology, Harvard University, 26 Oxford Street, Cambridge, MA 02138, USA

**Keywords:** gastropod phylogeny, transcriptomes, sequence heterogeneity, Psilogastropoda, Angiogastropoda, Mollusca

## Abstract

Gastropod molluscs are among the most diverse and abundant animals in the oceans, and are successful colonizers of terrestrial and freshwater environments. Past phylogenetic efforts to resolve gastropod relationships resulted in a range of conflicting hypotheses. Here, we use phylogenomics to address deep relationships between the five major gastropod lineages—Caenogastropoda, Heterobranchia, Neritimorpha, Patellogastropoda and Vetigastropoda—and provide one congruent and well-supported topology. We substantially expand taxon sampling for outgroups and for previously underrepresented gastropod lineages, presenting new transcriptomes for neritimorphs and patellogastropods. We conduct analyses under maximum-likelihood, Bayesian inference and a coalescent-based approach, accounting for the most pervasive sources of systematic errors in large datasets: compositional heterogeneity, site heterogeneity, heterotachy, variation in evolutionary rates among genes, matrix completeness, outgroup choice and gene tree conflict. We find that vetigastropods and patellogastropods are sister taxa, and that neritimorphs are the sister group to caenogastropods and heterobranchs. We name these two major unranked clades Psilogastropoda and Angiogastropoda, respectively. We additionally provide the first genomic-scale data for internal relationships of neritimorphs and patellogastropods. Our results highlight the need for reinterpreting the evolution of morphological and developmental characters in gastropods, especially for inferring their ancestral states.

## 1. Introduction

Gastropods are one of the most diverse clades of marine animals [1], and the only mollusc group to successfully colonize terrestrial environments. With an extant diversity of many tens of thousands of described species, gastropods also have a high degree of morphological disparity—snails, limpets and slugs with enormous variation in shell shape, coloration and size—and inhabit all kinds of environments and depths. Gastropods have embryonic spiral cleavage, an array of developmental modes (direct and indirect, with more than one type of larva), and undergo torsion of the body during development. Five main lineages are currently recognized: Caenogastropoda (e.g. cowries, whelks, conchs, cones), Heterobranchia (e.g. bubble snails, sea slugs, sea hares, most terrestrial snails and slugs), Neritimorpha (nerites), Patellogastropoda (true limpets) and Vetigastropoda (e.g. abalones, keyhole limpets, turban snails, top shells).

Early classifications included members of the vetigastropods, patellogastropods and neritimorphs in the Archaeogastropoda [2,3]. With the first numerical cladistic analysis of morphological data, patellogastropods were recovered as the sister group to all other gastropods, which were united in the clade Orthogastropoda [4,5]. The sister group relationship of the most diverse lineages, the heterobranchs and caenogastropods into the clade Apogastropoda, has been consistently recovered in most morphological and molecular analyses. Other than that, almost all possible topologies for gastropod relationships have been proposed (for a historical review, see [6]). Early molecular studies had mixed success in recovering even the well-established monophyly of gastropods or some of the main lineages [7–11]. Mitogenomic efforts have also produced discordant results [12–14], but recently have recovered a topology congruent with orthogastropods [15]. The first transcriptomic analyses of the group were able to reject several of the historically proposed hypotheses, including the clade Orthogastropoda [16]. However, different methods still resulted in contrasting topologies, and three hypotheses remain [16]. The major uncertainty is the position of Neritimorpha, which is recovered either as the sister group to Apogastropoda or as the sister group to Patellogastropoda and Vetigastropoda, in this case forming the traditional Archaeogastropoda. The third remaining hypothesis has vetigastropods as the sister lineage to all other gastropods [16].

Although the most diverse gastropod lineages were well sampled in the transcriptomic analyses of Zapata *et al.* [16], the dataset had only one species of Patellogastropoda and two of Neritimorpha, which are crucial for the proper rooting of the gastropod tree. As the three remaining hypotheses differ in their rooting, better outgroup sampling is another key necessary improvement. Furthermore, several biases known to be present in large genomic datasets have not been accounted for in the phylogenetic methods used so far to resolve gastropod relationships. Heterogeneity in the stationary frequency of amino acids among samples is one such issue that can artificially group taxa that are actually not closely related based on convergent amino acid composition [17]. Within-site rate variation through time (heterotachy) is another likely violation [18]. Some genes with slow rates of evolution (e.g. ribosomal protein genes) have also been shown to bias phylogenetic inference [19,20], while genes with fast rates and high levels of saturation can cause long-branch attraction [15,21]. An additional model violation comes from gene tree discordance, not accounted for by concatenation methods, that can be caused by incomplete lineage sorting and be particularly relevant in areas of the tree with short internal branches [22–24], such as the radiation of crown gastropods during the Ordovician [16,25]. More commonly considered issues include rate heterogeneity between sites and missing data.

Our goal was to resolve between the three remaining hypotheses for the early divergences of gastropods. We present an extended sampling of Neritimorpha and Patellogastropoda by producing new transcriptomes, and complement the dataset with the latest published gastropod transcriptomes. We further increase representation for the closest outgroups—bivalves, scaphopods and cephalopods—sampling all of the major lineages within each of these mollusc clades. We employ a variety of methods and models with strategic gene subsampling to account for the most widespread potential sources of systematic error in large datasets, namely compositional heterogeneity, site heterogeneity, heterotachy, variation in evolutionary rates among genes, matrix completeness, outgroup choice and gene tree conflict.

## 2. Methods

### (a) Sampling and sequencing

We sequenced the transcriptomes of 17 species, mostly patellogastropods and neritimorphs, and combined them with published transcriptome sequences from 39 other gastropods and 18 mollusc outgroups, for a total of 74 terminals. All new data and selected published sequences are paired-end Illumina reads. New samples were fixed in RNA*later* (Invitrogen) or flash frozen in liquid nitrogen. RNA extraction and mRNA isolation were done with the TRIzol Reagent and Dynabeads (Invitrogen). Libraries were prepared with the PrepX RNA-Seq Library kit using the Apollo 324 System (Wafergen). Quality control of mRNA and cDNA was done with a 2100 Bioanalyzer, a 4200 TapeStation (Agilent) and the Kapa Library Quantification kit (Kapa Biosystems). Samples were pooled in equimolar amounts and sequenced in the Illumina HiSeq 2500 platform (paired end, 150 bp) at the Bauer Core Facility at the Harvard University. New sequences were deposited in the NCBI Sequence Read Archive (BioProject PRJNA508436, SRA SRR8318344–SRR8318360); voucher information, library indexes and assembly statistics are available in electronic supplementary material, table S1.

### (b) Transcriptome assembly

Both new and previously published transcriptomes were assembled *de novo*; a detailed pipeline, scripts and assemblies are available in the electronic supplementary material. Raw reads were cleaned with RCorrector [26] and Trim Galore! [27], removing unfixable reads (as identified by RCorrector), Wafergen library adapters and reads shorter than 50 bp. Filtered reads were compared against a set of mollusc ribosomal RNAs and mitochondrial DNA and removed with Bowtie2 v. 2.2.9 [28]. This set was created from the well-curated databases SILVA [29] (18S and 28S rRNAs), AMIGA [30] (mtDNA) and from GenBank [31] (5S and 5.8S rRNAs), and is also deposited in the electronic supplementary material. Reads were assembled into transcripts with Trinity v. 2.3.2 [32,33] (–SS_lib_type FR for our new strand-specific data generated with Wafergen kits; precise information was not available from published data, so the default non-strand-specific mode was used for reads downloaded from SRA). A second run of Bowtie2 was done on the assemblies, before removing transcripts with sequence identity higher than 95% with CD-HIT-EST v. 4.6.4 [34,35]. Transcripts were then translated to amino acids with TransDecoder v. 3.0 [33], and the longest isoform of each gene was retained with a custom python script (*choose_longest_iso.py*). The completeness of the assemblies was evaluated with BUSCO v. 3.0.2 by comparison with the Metazoa database [36].

### (c) Matrix construction

**Figure 1.**
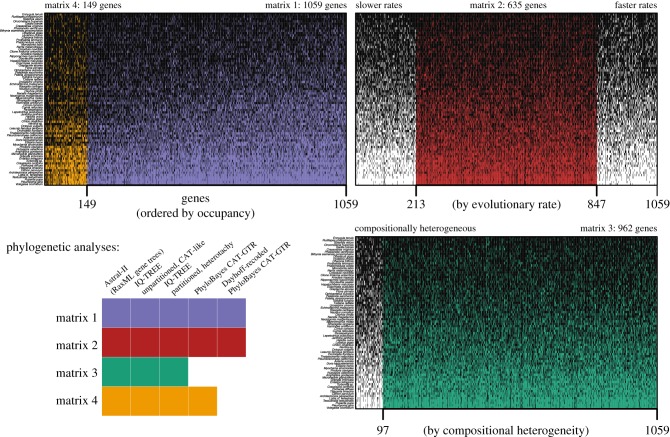
Matrices and phylogenetic methods used to infer gastropod relationships. With 50% taxon occupancy, matrix 1 is the largest, with 1059 genes. Matrix 4 is the subset of the best sampled 149 genes, with 70% taxon occupancy. Genes and species are sorted with the best sampling on the upper left. Matrix 2 is the subset of 635 genes after ordering all genes by evolutionary rate and removing the 20% slowest and 20% fastest evolving genes. Matrix 3 includes the 962 genes that are homogeneous in amino acid composition; genes are ordered by *p*-value of the homogeneity test. Black cells indicate genes present for each species. See Methods for details. (Online version in colour.)

We built four matrices to account for extreme evolutionary rates, amino acid composition heterogeneity and different levels of matrix completeness. Scripts, gene content for each matrix and alignment files are available in the electronic supplementary material. Orthology assignment of the peptide assemblies was done with OMA v. 2.0 [37]. We then used a custom python script (*selectslice.py*) to select all orthogroups for which at least half of the terminals were represented (50% taxon occupancy), resulting in a matrix with 1059 genes (matrix 1) (figure 1). Each orthogroup was aligned with MAFFT v. 7.309 [38], and the alignment ends were trimmed to remove positions with more than 80% missing data with a custom bash script (*trimEnds.sh*). To avoid possible biases, saturation and long-branch attraction, matrix 2 was built by removing from matrix 1 the 20% slowest and the 20% fastest evolving genes, as calculated with TrimAl [39], for a final size of 635 genes (figure 1). Matrix 3 is the subset of 962 genes from matrix 1 that are homogeneous regarding amino acid composition. Homogeneity for each gene was determined with a simulation-based test from the python package p4 [17,40], with a custom script modified from Laumer *et al.* [41] (*p4_compo_test.py*) and a conservative *p*-value of 0.1. Finally, a subset of 149 genes with 70% taxon occupancy constitutes matrix 4 (figure 1). For inference methods that require concatenation, genes were concatenated using Phyutility [42]. We further reduced composition heterogeneity in matrices 1 and 2 by recoding amino acids into the six Dayhoff categories [43] with a custom bash script (*recdayhoff.sh*).

All of our matrices include a dense outgroup sampling from the closest mollusc relatives. However, most previous molecular gastropod phylogenies have sampled only a couple outgroup species and/or very distantly related molluscs. To test the effect of such limited outgroup sampling, we built four extra datasets based on the largest matrix 1, each containing all gastropods plus only one of the other mollusc classes from our complete set (bivalves, scaphopods, cephalopods or polyplacophorans).

### (d) Phylogenetic analyses

Amino acid matrices were used for phylogenetic inference with a coalescent-based approach in Astral-II v. 4.10.12 [44], with maximum likelihood (ML) in IQ-TREE MPI v. 1.5.5 [45–47] and with Bayesian inference in PhyloBayes MPI v. 1.7a [48]. The two Dayhoff-recoded matrices were analysed in PhyloBayes (figure 1). Full details and scripts are explained in a custom pipeline in the electronic supplementary material. For the coalescent-based method, gene trees were inferred with RAxML v. 8.2.10 [49] (-N 10 -m PROTGAMMALG4X) and then used as input for Astral-II for species tree estimation. For each concatenated matrix, we inferred the best ML tree with two strategies: a gene-partitioned analysis with model search including LG4 mixture models and accounting for heterotachy (-bb 1500 -sp partition_file -m MFP+MERGE -rcluster 10 -madd LG4 M,LG4X -mrate G,R,E); and a non-partitioned analysis with model search also including the C10 to C60-profile mixture models [50] (ML variants of the Bayesian CAT model [51]) (-bb 1500 -m MFP+MERGE -rcluster 10 -madd LG4 M,LG4X,LG+C10,LG+C20,LG+C30,LG+C40,LG+C50,LG+C60 -mrate G,R,E). The search for the models LG+C60 (matrices 1 and 3) and LG+C50 (matrix 1) required more memory than available, and these models were disregarded for the respective matrices. Outgroup test datasets were analysed with the ML profile mixture model. PhyloBayes was run with the CAT-GTR model on a subset of the concatenated alignments (matrices 1, 2 and 4), discarding constant sites to speed up computation. Tree figures were edited with the R package *ggtree* [52].

## 3. Results and discussion

### (a) Main gastropod relationships

**Figure 2.**
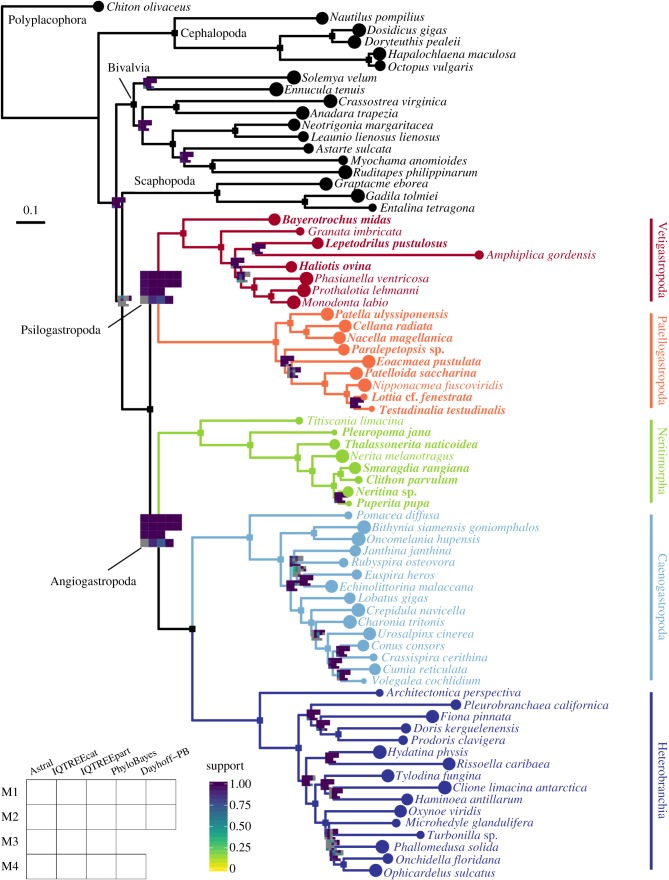
Gastropod phylogeny inferred from the largest matrix (M1) with ML and a profile mixture model (IQTREEcat). A single square marks branches where all analyses had full support; branches where at least one analysis had less than full support are marked with a plot, coloured in a continuous scale according to support value, from 0 to 1. Grey squares in the plots represent splits that were absent in a given analysis. New transcriptomes are represented in bold. M1–M4, matrices 1–4; IQTREEpart, ML partitioned analysis; Dayhoff-PB, Bayesian analysis on a matrix recoded according to the six Dayhoff categories. See Methods for details. (Online version in colour.)

Our main goal was to resolve the deep nodes of the gastropod tree and distinguish between three hypotheses of the relationships among its five main lineages. All but one of our inference methods and matrices congruently support a clade uniting Vetigastropoda with Patellogastropoda, and Neritimorpha as the sister group to Apogastropoda (figure 2). The only exception is the coalescent-based analysis on the smallest dataset of 149 genes (Astral, matrix 4), in which these two key nodes were left unresolved (all tree files are available in the electronic supplementary material). Accordingly, the few analyses with lower support on these nodes also refer to the smaller matrix 4, which is unsurprising given that it comprises fewer informative sites in concatenated analyses and fewer genes in the coalescent-based analysis [53]. In summary, the resulting topology is congruent based on an array of analyses testing for the major common sources of systematic error in phylogenomic datasets, including gene tree discordance, compositional heterogeneity, heterotachy, site heterogeneity, variation in evolutionary rates and missing data.

To explore the signal of genes with heterogeneous amino acid composition, we used the ML and coalescent-based methods to infer trees for the set of 97 genes that failed the p4 homogeneity test (trees available in the electronic supplementary material). Interestingly, in the ML partitioned analysis that had a simpler site heterogeneity model, Patellogastropoda was recovered as the sister group to all other lineages. This is possibly the most commonly used strategy for phylogenetic inference, highlighting the risks of not accounting for high complexity in sequence data, in this case, site and composition heterogeneity combined. Even with exclusively heterogeneous genes, analyses that do not rely on a concatenated matrix (coalescent-based) or that consider more complex models of site heterogeneity (ML with a profile mixture model) still recovered the same relationships of figure 2.

Our enriched taxon representation ensured that all major lineages within each of the closest outgroups (scaphopods, bivalves and cephalopods) were represented, mitigating issues of long-branch attraction to the outgroups (figure 2). However, most previous molecular studies of gastropods had limited outgroup representation, often resulting in long branches leading to the ingroup [9,10,14,15]. We tested the sensitivity of our results to restricted outgroup sampling by limiting outgroups to just one mollusc class at a time in matrix 1 (trees available in the electronic supplementary material). Datasets with only cephalopods, only bivalves and even with the single polyplacophoran resulted in the same topology of figure 2. Only the dataset restricted to scaphopods produced a different topology, finding patellogastropods as the sister group to all other gastropods, but with low support. Scaphopods and patellogastropods have respectively the longest internal branch among outgroups and gastropods, pointing to an effect of long branch attraction. These results highlight the importance of maximizing outgroup sampling when targeting hard and ancient nodes.

Our inferred topology for gastropod relationships (figure 2) has been previously recovered by a few molecular [16,54] and total evidence [6] analyses, with numerous alternatives proposed in the literature (e.g. [5,6,10,12,13,15,53]), even within the same studies. With 17 analyses (combinations of four subsampled matrices, two data types—amino acids and Dayhoff recoding—and four inference methods/models), for the first time, we find strong congruence towards this single topology for deep gastropod relationships. With that we reject the clade Archaeogastropoda, proposed almost a century ago by Thiele [2], which united Neritimorpha, Vetigastropoda and Patellogastropoda. Although this grouping had given way to other predominant hypotheses along the years (e.g. Eogastropoga versus Orthogastropoda divergence), this classification is still used in the organization of malacology and paleontology collections of many natural history museums, and was one of the three resulting hypotheses from the transcriptomic study of Zapata *et al.* [16].

The close relationship of neritimorphs and apogastropods had already been recognized based on early developmental characters [55,56], such as the time of formation of the 4d blastomere (mesentoblast). In these groups, the differentiation of this key embryonic cell, which gives rise to the mesoderm in spiralians [57–59], is accelerated, happening at an earlier cell stage than in vetigastropods and patellogastropods [55,56]. Other traits shared by neritimorphs and apogastropods include complex reproductive anatomy, internal fertilization and encapsulated eggs, which hatch into a feeding veliger larva or directly into a juvenile [6,25,60–62]. By contrast, vetigastropods and patellogastropods are mostly broadcast spawners, with embryos that develop in the plankton into non-feeding larvae, first as a trochophore that later gives rise to a veliger [6,25,60,62,63]. Character states shared by patellogastropods and vetigastropods have historically been interpreted as plesiomorphic based on the phylogenetic hypothesis in which patellogastropods were the sister group to all other gastropods, or due to a misguided notion that these are ‘primitive’ taxa [5,55]. In the light of a sister group relationship between Patellogastropoda and Vetigastropoda, it is not possible to confidently infer which were the ancestral gastropod conditions without an extensive comparative analysis. Sampling of morphological and developmental data from a larger diversity of gastropods and especially their closest outgroups—bivalves and scaphopods—will be needed to reinterpret their evolution under the framework presented here (figure 2).

Although exceptions exist in such diverse clades, we use the most general features of the reproductive strategy and early life history of gastropods, irrespective of their ancestral state, to name the two major lineages in figure 2: Psilogastropoda, **new taxon**, from the Greek *psilos* meaning bare, naked. This is the most inclusive clade containing Vetigastropoda and Patellogastropoda, but not Neritimorpha, Caenogastropoda or Heterobranchia, therefore also accounting for stem taxa. The name represents the unprotected nature of the gametes, which are released in the water for external fertilization, and of the embryos and larvae that develop in the plankton, exposed to the environment. Angiogastropoda, **new taxon**, from the Greek *angeion* meaning vessel, capsule. It is the most inclusive clade containing Neritimorpha, Caenogastropoda and Heterobranchia, but not psilogastropods. The name reflects the enclosed nature of the embryo after internal fertilization, which is encapsulated during early development, followed by either direct development or a late stage veliger larva hatching from the egg.

We then propose the adjusted classification of Gastropoda (table 1). Important questions that remain regarding major gastropod relationships include the position of Cocculiniformia and Neomphalina, smaller deep sea clades that have been considered somehow related to vetigastropods, neritimorphs, patellogastropods or as independent branches in the gastropod tree [60]. They are yet to be sampled in a phylogenomic analyses, and we therefore keep their independent status relative to the other major lineages, but note that future phylogenomic studies could reveal either one as part of psilogastropods or angiogastropods.

**Table 1.** Higher level classification of the extant Gastropoda proposed here. We follow [64] in not presenting the authority of high level names because some of them have a taxonomic composition that differs substantially from that of the original author.

classification proposed here
Class Gastropoda
** Psilogastropoda**, new taxon
** **Patellogastropoda
** **Vetigastropoda
** Angiogastropoda**, new taxon
** **Neritimorpha
** **Apogastropoda
** **Caenogastropoda
** **Heterobranchia
** ***Incertae sedis*
** **Neomphalina
** **Cocculiniformia

Regarding overall mollusc relationships, we recover a well-supported clade of gastropods, bivalves and scaphopods in all analyses; however, as in previous phylogenomic efforts [65,66], relationships between these three groups are unstable (figure 2). The Dayhoff datasets and most of the ML analyses with the profile mixture model result in a clade of gastropods and scaphopods; while most coalescent-based trees recover a clade of bivalves and scaphopods; and finally, the ML partitioned analyses produce a clade of gastropods and bivalves. Perhaps a way ahead to resolve such hard nodes will be to use other types of data, such as genomic rearrangements and presence/absence of genes from complete genomes.

### (b) A note about convergence in PhyloBayes

While PhyloBayes runs converged on the Dayhoff-recoded datasets presented here, analyses on the more complex amino acid matrices did not converge for all parameters. The problem was especially pronounced for the large matrices (a summary table with convergence metrics for all analyses is given in the electronic supplementary material). We observed that some convergence issues were due to small differences between chains regarding the position of one or few derived terminals within the outgroups or within apogastropods, whose relationships were not the goal of this study. We suspect this may be caused by a problem in topology proposals for these derived nodes, leading some of the chains to get stuck in local maxima. One example comes from the Dayhoff analysis of matrix 1: the initial two chains seemed to be very far from topological convergence (maxdiff = 1) even after more than 20 000 generations. Upon closer inspection, both trees were basically indistinguishable, with the only variation being the position of *Charonia* or *Crepidula* as the sister group to Neogastropoda. Removal of either one of the two terminals from the treelist files with a custom script (*remove_terminal_treelist.py*) resulted in the same converged topology (tree files in the electronic supplementary material). For that particular analysis, we ran two additional independent chains that converged without presenting this issue. This behaviour was recently discussed [67], and perhaps has been underreported in the literature.

### (c) Relationships within gastropod lineages

This is the first genomic-scale dataset for Patellogastropoda and Neritimorpha. Internal relationships of patellogastropods have presented incongruent results even among studies using the same type of data (reviewed in [60,68]). We consistently recover Nacellidae (*Cellana*, *Nacella*) as the sister group of Patellidae (figure 2), a clade originally supported by some of the earliest morphological [69] and mitochondrial phylogenies [70]. Nacellids have also been placed either as a grade at the base of the tree [71] or closer to Lottiidae [72], and the current taxonomic classification has Nacellidae in the superfamily Lottioidea [64,73]; our results indicate the family should be transferred to Patelloidea. Another interesting finding regards *Eoacmaea*, which had gained family and superfamily status due to being recovered as the sister taxon to all other patellogastropods with mitochondrial markers [72]. None of our results recover this position, but rather indicate that the genus is either part of Lottiidae (most ML and Bayesian results), which was its original assignment, or is sister group to the Lottioidea families Neolepetopsidae (*Paralepetopsis*) and Lottiidae (*Patelloida*, *Nipponacmea*, *Lottia*, *Testudinalia*) (coalescent-based trees and one ML tree) (figure 2).

Neritimorphs had mostly congruent phylogenies recovered from 28S rRNA [74] and mitogenomes [75]. Our reconstruction supports the same topology, with Neritopsoidea (*Titiscania*) as the sister group to all other neritimorphs, followed by the divergence between Helicinoidea (*Pleuropoma*) and Neritoidea (figure 2). Within the latter, we recover a monophyletic Neritidae as the sister group of Phenacolepadidae (*Thalassonerita*). The nested position of *Smaragdia* inside Neritininae disagrees with the current classification of the genus in its own subfamily [64,73].

Vetigastropoda and Heterobranchia had similar taxon representation as in Zapata *et al.* [16] (with newly sequenced replacements for some vetigastropod families). As expected, the relationships are the same, and highlight the need for future studies focused on each group, given the uncertain position of *Haliotis* in Vetigastropoda, and low resolution of internal relationships of panpulmonates in Heterobranchia (figure 2). Our results contrast with recent mitogenomic analyses of vetigastropods, which recovered a monophyletic group of Seguenzioidea (*Granata*), Lepetodriloidea (*Lepetodrilus*) and Haliotoidea (*Haliotis*) [14,76,77].

We substantially increased sampling of Caenogastropoda by adding the latest published transcriptomes of eight families. Despite that, caenogastropods are the most diverse gastropod lineage, with over a hundred families, and the following results are still limited in sampling. We recover a monophyletic Neogastropoda; its internal relationships differ from a molecular study with denser taxon sampling [78], in that we find Buccinoidea (*Cumia*, *Volegalea*) closer to Conoidea (*Conus*, *Crassispira*) than to Muricoidea (*Urosalpinx*). We also recover a monophyletic Truncatelloidea (*Bithynia*, *Oncomelania*) as the sister group to all other Hypsogastropoda (figure 2). The relative position of Tonnoidea (*Charonia*) and Calyptraeoidea (*Crepidula*) regarding Neogastropoda is unclear; nonetheless, the close relationship between Tonnoidea, Neogastropoda and also Stromboidea (*Lobatus*) agrees with previous molecular studies [78,79]. The branching pattern of the closest relatives of neogastropods reveals a paraphyletic Littorinimorpha.

## Data accessibility

New transcriptomes were deposited in the NCBI Sequence Read Archive (BioProject PRJNA508436). Electronic supplementary materials are deposited in Harvard Dataverse: https://doi.org/10.7910/DVN/O85KLQ. Scripts are available from: https://github.com/tauanajc/Cunha_Giribet_2019_ProcRSocB.
